# Effect of Coconut Oil Pulling and Benzydamine Hydrochloride Mouthwash in the Management of Radiation-Induced Oral Mucositis: a Randomized Controlled Clinical Trial

**DOI:** 10.1007/s12663-025-02515-2

**Published:** 2025-03-20

**Authors:** Nancy Agarwal, Premalatha Shetty, Sameep Shetty, B. Dhanashree, K. M. Sandeep

**Affiliations:** 1 Department of Oral and Maxillofacial Surgery, Manipal College of Dental Sciences Mangalore, Manipal Academy of Higher Education, Manipal, Karnataka 576104 India; 2 Department of Microbiology Kasturba Medical College Mangalore, Manipal Academy of Higher Education, Manipal, Karnataka 576104 India

**Keywords:** Radiotherapy, Oral mucositis, Coconut oil, Oil pulling, Candida, Benzylamine hydrochloride, Health and well-being

## Abstract

**Introduction:**

Oral mucositis is a painful and debilitating inflammation of the oral mucosa, commonly occurring as an acute side effect of radiation therapy in patients with head and neck cancer. The condition is often aggravated by secondary infections, such as candidiasis, complicating its management. Although coconut oil has shown promising therapeutic properties, particularly through the practice of oil pulling, its role in the treatment or prevention of radiation-induced oral mucositis remains clinically unexamined.

**Method:**

A total of 127 individuals with clinically and histopathologically confirmed oral malignancies, scheduled for radiation therapy, were selected from the Radiotherapy and Oncology Department of a tertiary care centre in Mangalore. The study compared two interventions: coconut oil pulling in one group and benzydamine hydrochloride mouthwash (Tantum) in the other. Swab samples were collected before and after the study to assess the reduction in candida growth. Patients were evaluated biweekly for mucositis, pain, and trismus. At the end of the trial, participants completed a quality of life questionnaire to further assess the outcomes.

**Results:**

The coconut oil group demonstrated a statistically significant reduction in the total colony count of Candida species from pre to post-treatment (*p* = 2.3 × 10^–39^). Additionally, there was a notable delay in the onset and a reduction in the severity of mucositis in this group (*p* = 0.0000002). Significant improvements were observed in pain (*p* < 0.001), and trismus (*p* = 0.006) alongside an overall enhancement in the quality of life (*p* = 0.009).

**Conclusion:**

Coconut oil pulling exhibited significant antifungal, anti-inflammatory, and analgesic properties, effectively reducing and preventing radiation-induced oral mucositis and its associated complications.

## Introduction

Head and neck cancer, particularly squamous cell carcinoma, ranks among the most prevalent cancers due to its high morbidity and the significant consequences of treatment. Oral carcinoma is the sixth most frequent cancer globally, accounting for 4–5% of all cancer cases. It has a male-to-female ratio of 4:1 and is more prevalent in individuals over 40 years of age [1].

Treatment modalities for head and neck cancers include surgery, chemotherapy, and radiotherapy. While surgery has traditionally been the cornerstone of oral carcinoma management, radiation therapy has proven particularly effective for treating head and neck malignancies, either as a primary treatment or as an adjuvant to other therapies. However, radiation is associated with both transient and permanent adverse effects, including ageusia, xerostomia, and mucositis, which significantly impact patient quality of life [1–3].

Oral mucositis (OM) is a severe complication of radiation therapy for head and neck cancers. It manifests as erythematous and ulcerative lesions of the oral mucosa. Besides being painful, OM adversely affects nutritional intake, oral hygiene, and infection susceptibility, often requiring additional care measures like total parenteral nutrition [4]. OM frequently interrupts cancer treatment schedules, potentially leading to tumour progression or recurrence, thereby directly influencing prognosis. As a dose-limiting side effect of radiation therapy, OM remains a critical challenge in cancer rehabilitation [5, 6].

The pathogenesis of radiation-induced mucositis involves five stages: initiation, message generation, signal amplification, ulceration, and healing. Radiation damages basal epithelial cells, triggering the overexpression of pro-inflammatory cytokines such as TNF-α, IL-1, and IL-6. This cytokine cascade increases local vascularization, leading to inflammation and ulceration. Secondary infections, particularly fungal infections like candidiasis, exacerbate the severity of OM by further upregulating pro-inflammatory cytokines. During the healing phase, epithelial cells regenerate to repair damaged tissues. However, opportunistic bacterial colonization of ulcerated sites often complicates this process [1].

Various treatment approaches, both local and systemic, have been explored to manage radiation-induced OM. These include antibiotics, antiseptics, anti-inflammatory drugs, honey, vitamins, antioxidants, amino acids, cryotherapy, and low-level laser therapy [7, 8]. While these modalities aim to reduce secondary infections and control inflammation, no universally effective prophylactic or curative treatment has been established [9].

Benzydamine hydrochloride, a non-steroidal agent, exhibits antimicrobial, anti-inflammatory, topical analgesic, and anaesthetic properties. It has shown efficacy in treating OM in head and neck cancer patients undergoing radiation therapy and is commercially available as Tantum mouthwash [10].

The potential role of plant-based therapies, particularly essential oils, has also been investigated for their anti-inflammatory, analgesic, and antimicrobial properties. However, the specific benefits of essential oils in radiation-induced mucositis remain largely unexplored, warranting further research into their therapeutic potential.

## Methodology

The study was conducted between November 2017 and September 2019 in the Departments of Oral and Maxillofacial Surgery and Oncology and Radiotherapy at a tertiary care centre in Mangalore. Patients undergoing radiation therapy for head and neck cancer who provided informed consent were included in the study.

Exclusion criteria included patients with pre-existing mucositis, those taking additional medications to prevent mucositis, individuals physically unable to rinse an oral solution for the required duration, and patients with cognitive impairments or lack of motivation to participate in the trial.

A total of 127 individuals with clinically and histopathologically confirmed cases of oral cancer scheduled for radiation therapy were selected. After obtaining informed consent, participants were randomly assigned to two groups using a random sampling technique. Group A (treatment arm) consisted of 64 subjects who were instructed to perform coconut oil pulling, while Group B (control arm) included 63 subjects who used Tantum oral rinse.

This was a double-blind study, ensuring that both participants and the operator were unaware of the group assignments to eliminate potential bias.

## Technique

Prior to the commencement of the investigation, all subjects in both trial arms underwent a thorough intraoral examination to exclude any pre-existing mucositis. Following this, participants were assessed biweekly to monitor the development of oral mucositis.

A swab from tongue was collected from each participant in both groups one day before initiating radiation therapy. The procedure involved gently swabbing the tongue with a sterile cotton swab, after which the samples were transported to the microbiology laboratory for analysis.

The samples were streaked onto a primary isolation medium, such as Sabouraud's dextrose agar (SDA), and incubated aerobically at 37 °C for 24 to 48 h. Candida growth was identified by the appearance of smooth, cream- or white-coloured, yeast-like colonies. If no growth was observed after 72 h of incubation, the culture was considered negative.

Patients in Group A were instructed to use 15 ml of pure coconut oil starting one day prior to radiation therapy. They were advised to swish the oil slowly around their mouth for 15 min, ensuring thorough contact with the oral mucosa, before spitting it out. This process was repeated at specific intervals: 15 min before radiation therapy, 15 min after radiation therapy, 2 h post-therapy, and 6 h post-therapy.

Participants in Group B were directed to use 15 ml of an undiluted mouthwash containing 0.15% benzydamine hydrochloride one day prior to radiotherapy. They were instructed to rinse for at least five minutes, ensuring adequate contact with the oral mucosa, before spitting it out. The rinsing protocol was the same as Group A: 15 min before radiation, 15 min after radiation, 2 h post-therapy, and 6 h post-therapy.

Patients in the coconut oil group who developed grade III mucositis during the course of therapy were withdrawn from the trial and provided standard hospital care, which included the application of topical lignocaine gel.

Four weeks after completing radiation therapy, all patients were monitored. They were asked to complete a quality of life (QOL) questionnaire to assess various aspects of their well-being, including overall health, pain levels, and difficulties with swallowing.

Evaluation was done using the RTOG mucositis assessment scale; based on which grading was done. The day which marks the onset of mucositis (grade 1), as well as its progression to different grades, were noted the onset of mucositis (Grade 1) and its progression through subsequent grades were meticulously noted. The severity of pain and trismus were evaluated using VAS and RTOG scale respectively. VAS scores from 1–3 were considered as grade 1, 4–6 was considered as grade 2 and 7–10 was considered as grade 3.

## Results

Out of 127 patients, 64 were assigned to the treatment arm, whereas 63 were assigned to the control arm. One patient from the control arm was dropped out due to withdrawal of consent, whereas two patients in the treatment arm dropped out due to the severity of symptoms of mucositis. (Grade 3) Remaining 124 patients were included in the study.

**Fig. 1:**
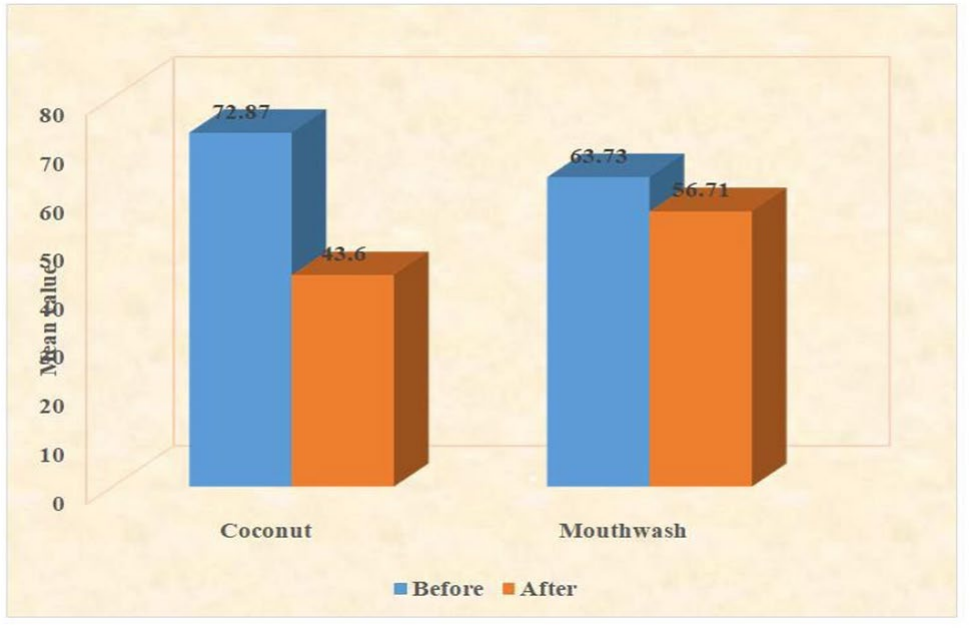
Comparison of candida colony count in both the groups

The number of Candida colonies in each group, both before and after the study's conclusion, is shown in Table 1 and Fig. 1. The t-value for the control arm was 2.29, while it was (*p* = 19.328) in the treatment arm. A highly significant reduction (*p* = 2.3 × 10^–39^) in the number of Candida colonies was observed in the treatment arm when comparing pre- and post-study results.

**Table 1:** Comparison of candida colony in both the groups

Group	Candida colony count before the study	Candida colony count after study	Reduction in colony count	*t* value	*P*-value
Treatment	72.87	43.60	29.27	19.328	2.3 × 10^–39^
Control	63.73	56.71	7.016	2.29	0.0236

**Fig. 2:**
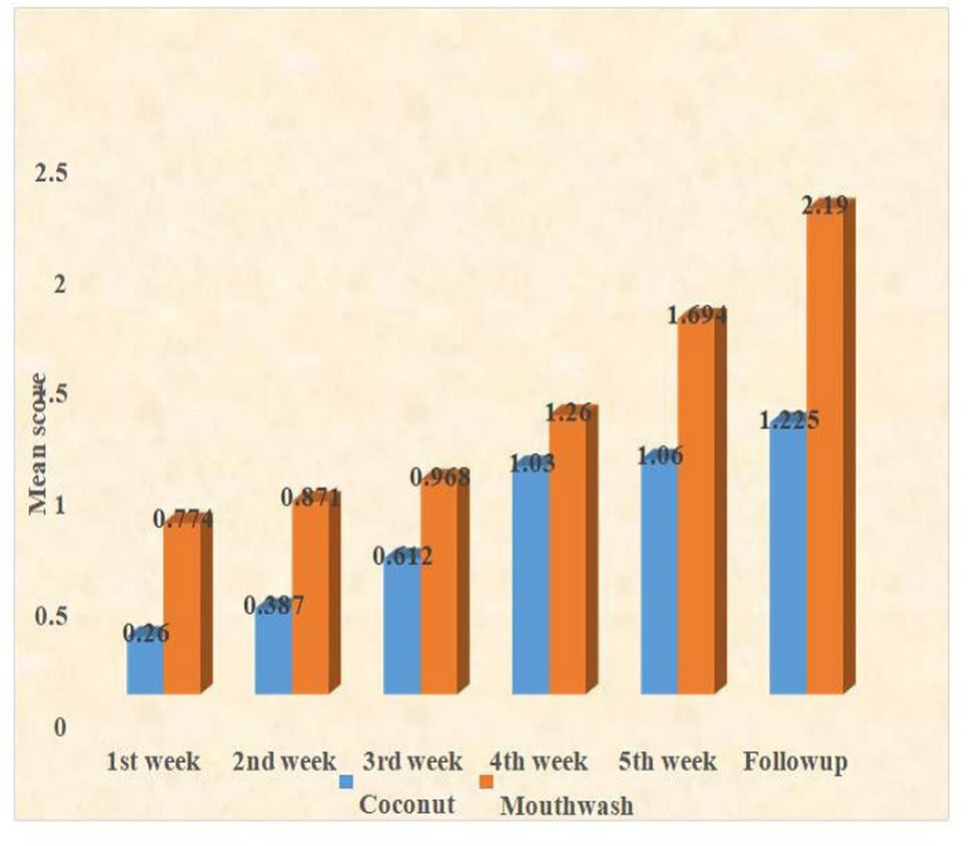
Mean mucositis score at the end of each week

The mean mucositis scores for both groups, recorded at weekly intervals throughout the study, are shown in Table 2 and Fig. 2. At baseline, neither group exhibited any mucositis scores. By the end of the first week, the control group had a mean score of 0.774, while the test group's mean score remained at zero. During the second week, the test group's mean score increased to 0.387, compared to 0.871 in the control group. This trend continued, with the test group's mean score reaching 1.225 at follow-up, as opposed to 2.19 in the control group. A statistically significant difference in mucositis scores was identified between the two groups (*p* = 0.0000002). The progression of mucositis across weekly intervals for both groups is shown in Table 3. While most consecutive weeks did not show statistically significant differences in mucositis scores, notable changes were observed in the control group between the first and second weeks (*p* = 0.002) and between the third and fifth weeks (*p* = 0.001). In contrast, the test group did not exhibit statistically significant changes in mucositis scores over the same periods. The slower progression of mucositis in the test group compared to the control group was evident.

**Table 2:** Mean mucositis scores at the end of each week

	Treatment mean +/SD	Control mean + /−SD	*t* test	*P* value
Baseline	0	0		
1st week	0	0.774 ± 0.575	10.758	0.000009 vhs
2nd week	0.387 ± 0.145	0.871 ± 0.212	15.038	0.0000005 vhs
3rd week	0.612 ± 0.599	0.968 ± 0.659	3.187	0.002 hs
4th week	1.03 ± 0.482	1.26 ± 0.505	2.626	0.0097 vhs
5th week	1.06 ± 0.582	1.694 ± 0.585	6.122	0.00002 vhs
6th week	0.365 ± 0.31	0.963 ± 0.312	10.834	0.000008 vhs
Follow up	1.225 ± 0.412	2.19 ± 0.508	11.766	0.0000002 vhs

**Table 3:** Comparison of the progression of mucositis in the two groups

Parameter			Group					
			Test group			Control group		
			Wilcoxon signed ranks test *Z* value	*p*-value		Wilcoxon signed ranks test *Z* value	*p*-value	
Mucositis	1st week	2nd week	1.73	.083	NS	3.16	.002	Sig
		3rd week	3.00	.003	Sig	3.22	.001	Sig
		4th week	3.28	.001	Sig	3.36	.001	Sig
		5th week	3.22	.001	Sig	3.28	.001	Sig
		6th week	3.18	.001	Sig	3.18	.001	Sig
		Follow up	3.18	.001	Sig	3.18	.001	Sig
	2nd week	3rd week	2.45	.014	NS	2.24	.025	NS
		4th week	3.32	.001	Sig	3.36	.001	Sig
		5th week	3.21	.001	Sig	3.18	.001	Sig
		6th week	3.15	.002	Sig	3.15	.002	Sig
		Follow up	3.15	.002	Sig	3.15	.002	Sig
	3rd week	4th week	2.24	.025	NS	2.83	.005	NS
		5th week	2.45	.014	NS	3.32	.001	Sig
		6th week	3.32	.001	Sig	3.36	.001	Sig
		Follow up	3.32	.001	Sig	3.36	.001	Sig
	4th week	5th week	1.00	.317	NS	1.73	.083	NS
		6th week	2.45	.014	NS	2.24	.025	NS
		Follow up	2.45	.014	NS	2.24	.025	NS
	5th week	6th week	2.24	.025	NS	1.41	.157	NS
		Follow up	2.24	.025	NS	1.41	.157	NS
	6th week	Follow up	2.24	.025	NS	1.41	.157	NS

Table 3 shows the progression of pain from a weekly basis in both groups. In the control group, a statistically significant change in pain scores was noted (*p*=.001) between the 1st and 3rd weeks’ observation. The test group, on the other hand, showed the first significant change in pain score (*p*=.001) only by the 5th week. The control group had a significant change in pain scores (*p*=.003) even from the 2nd week to 4th week.

This lack of significant score change in the test group on a weekly basis, when compared to the controls, points towards a slower rate of progression of pain in the test group (Table 4).

**Table 4:** Comparison of the progression of pain in the two groups

Parameter			Group					
			Test group			Control group		
			Wilcoxon signed ranks test *Z* value	*p*-value		Wilcoxon signed ranks test *Z* value	*p*-value	
Pain	1st week	2nd week	.00	1.000	NS	2.83	.005	NS
		3rd week	2.00	.046	NS	3.21	.001	Sig
		4th week	2.83	.005	NS	3.15	.002	Sig
		5th week	3.32	.001	Sig	3.18	.001	Sig
		6th week	3.36	.001	Sig	3.28	.001	Sig
	2nd week	3rd week	2.00	.046	NS	2.00	.046	NS
		4th week	2.83	.005	NS	3.00	.003	Sig
		5th week	3.32	.001	Sig	3.21	.001	Sig
		6th week	3.36	.001	Sig	3.28	.001	Sig
	3rd week	4th week	2.00	.046	NS	2.24	.025	NS
		5th week	2.65	.008	NS	2.83	.005	NS
		6th week	3.00	.003	Sig	3.16	.002	Sig
	4th week	5th week	1.73	.083	NS	1.73	.083	NS
		6th week	2.24	.025	NS	2.24	.025	NS
	5th week	6th week	1.41	.157	NS	1.41	.157	NS

Table 5: shows the difference in the overall pain score in both groups by calculating the number of tablets taken and VAS. The total number of analgesics consumed by the subjects in the treatment arm is 33 whereas 62 tablets have been taken by the subjects in the control arm. The mean of overall pain score in the treatment group is 4.85 and in the control arm, it is 7.48. So, the difference in the overall pain score in both groups is very highly significant (*p *= <0.001).

**Table 5:** Overall comparison of Analgesic consumed and Pain score in both the groups

	Group	*N*	Mean	Std. deviation	*t*
No. of tab	Treatment	33	3.48	1.839	12.189
	Control	62	10.00	2.758	*p* < 0.001 vhs
Pain score	Treatment	62	4.85	1.716	10.043
	Control	92	7.48	1.141	*p* < 0.001 vhs

Table 6 shows the difference in the quality of life in both the study groups. 82.2 % of the subjects in the treatment arm had a good quality of life as compared to the control group which had only 61.3%. In the control group, 38.7% reported with bad quality of life, whereas only 17.8% of the test subjects reported a bad quality of life according to the questionnaire. The overall difference between the two groups is significant statistically (*p *= 0.009).

**Table 6:** Comparison of the quality of life in both the groups

Group	No. of good quality of life	No. of bad quality of life	% of good quality of life	% bad quality of life	*P*-value of good quality of life
Treatment	51	11	82.2	17.8	*P* = 0.009 vhs
Control	38	24	61.3	38.7	

*X*2 = 6.727

## Discussion

Radiotherapy for head and neck cancer frequently causes tissue damage within the direct radiation portals, affecting the oral cavity, salivary glands, and taste buds. Oral mucositis is the most common and severe complication of cytotoxic cancer treatment, sometimes even life-threatening. In patients receiving radiation therapy for head and neck cancers, it is the primary dose-limiting side effect, often disrupting treatment or causing premature termination. These interruptions can hinder effective tumour control and negatively impact patient survival outcomes [11].

Coconut oil, also known as copra oil, is an edible oil extracted from the pulp or kernel of mature, dried coconuts harvested from the coconut palm (*Cocos nucifera*). It is predominantly composed of saturated fatty acids (SFA), which make up approximately 90% of its total composition. Mucositis, a major side effect of radiation therapy, has been effectively mitigated through the use of various medications and substances, including coconut oil.

To date, no technique has completely prevented mucositis; however, many have proven effective in reducing its severity and associated discomfort. Coconut oil, renowned for its numerous health benefits—including antimicrobial, anti-inflammatory, antifungal, anticancer properties, and its role in wound healing—has been widely recognized by researchers over the past few decades for its therapeutic potential. Due to these favourable attributes, coconut oil shows promise in preventing mucositis, alleviating discomfort, reducing trismus, and combating fungal infections during radiation therapy.

The findings of this study align with those of Ogbolu et al., who demonstrated that at a minimum inhibitory concentration (MIC) of 25%, coconut oil exhibited 100% sensitivity in Candida species susceptibility, compared to 88% sensitivity for fluconazole. They also noted that fluconazole was more fungistatic than fungicidal, even at higher doses, while in-vitro tests showed some fungicidal activity of capric acid and lauric acid, evidenced by decreased infectivity titre [13].

Similarly, a study by Shino et al. investigated the antibacterial efficacy of probiotics, ketoconazole, coconut oil, and chlorhexidine against *Candida albicans* isolated from young patients with early childhood caries. The study highlighted coconut oil's antifungal effectiveness, which was found to be comparable to ketoconazole and superior to probiotics in its advanced antifungal activity against *Candida albicans* [14]. Additionally, Bergsson et al. demonstrated the susceptibility of *Candida albicans* to various fatty acids and their 1-monoglycerides [15].

**Fig. 3:**
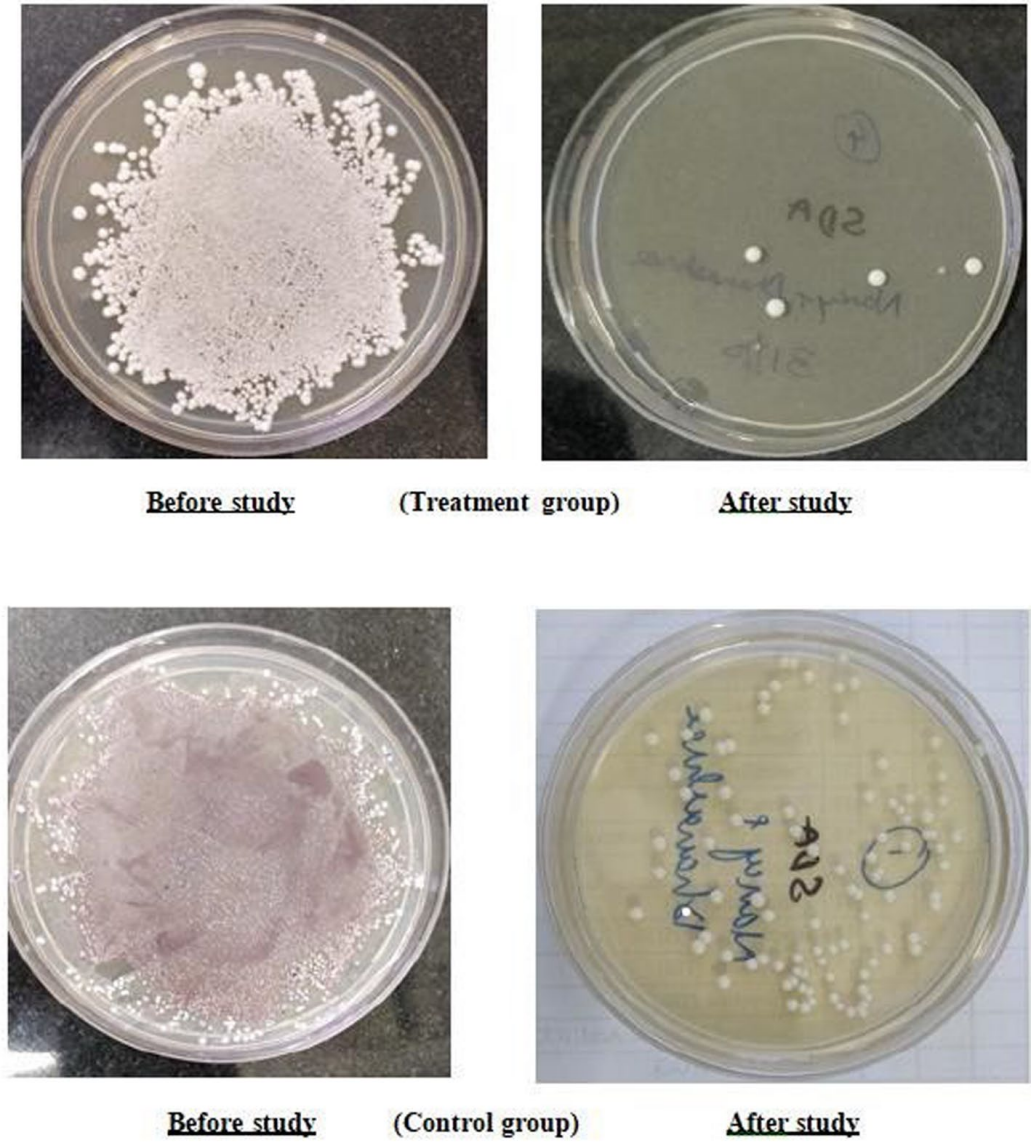
SDA plate showing candida colony culture before and after study

In our study, we observed a significant reduction in the number of *Candida* colonies following coconut oil pulling (*p* < 0.001, highly significant) (Fig. 3). Notably, mucositis onset was delayed in the test group compared to the control group. By the end of the first week of radiation therapy (1000 cGy), half of the control group had developed grade 1 mucositis, progressing to grade 2 by the end of the second week (2000 cGy). In contrast, no mucositis was observed in the test group during the same period.

By the third week (3000 cGy), all control group subjects had mucositis, with 75% advancing to grade 3 by the fourth week (4000 cGy). Conversely, mucositis in the test group became apparent only by the sixth week (6000 cGy), with only two-thirds of patients reaching grade 2 by the end of the treatment. Remarkably, several subjects in the test group did not develop mucositis at any point during the therapy (*p* = 0.001). The peak severity of mucositis was significantly lower in the test group compared to the control group.

These results are consistent with the findings of Mustafa et al., who observed that the treatment group receiving zinc sulfate had a median radiation therapy (RT) dose of 3600 cGy (3½ weeks) at the onset of mucositis, compared to 2000 cGy (2 weeks) in the control group. In their study, mucositis first appeared at 1800 cGy during the second week, peaked at the start of the third week, and reached its maximum severity by the middle of the fourth week before gradually declining. The difference in mean mucositis scores between the treatment and control groups became statistically significant after 2400 cGy (2½ weeks) and remained significant up to six weeks post-treatment [17].

Our findings also corroborated those of Karbassi et al., who demonstrated that patients in the experimental group treated with propolis experienced delayed mucositis onset and significantly reduced severity compared to the placebo group. The experimental group exhibited milder symptoms, with overall moderate mucositis severity [16].

During oil pulling, lauric acid in coconut oil reacts with sodium hydroxide present in saliva to form sodium laureate, which likely plays a key role in its anti-inflammatory effects by preventing bacterial adhesion to soft tissues. Additionally, the antioxidant properties of lauric acid help protect the oral mucosa from infection and inflammation [17]. Asokan et al. further highlighted that the emulsification of oil begins within five minutes of initiating oil pulling therapy, which contributes to its therapeutic benefits [18].

The mechanism of coconut oil pulling therapy, as explained by Peedikayil et al., involves the mechanical shear forces applied during swishing, which emulsify the oil and significantly increase its surface area. This process enables the oil to coat the tissue surfaces, reducing bacterial co-aggregation [17].

Our study demonstrated a significant delay in the onset of mucositis in the coconut oil group (3rd week, 19th–23rd day) compared to the benzydamine mouthwash group (1st week, 4th–7th day) (*p* < 0.001). The severity of mucositis was also markedly lower in the coconut oil group, with 66.1% of participants experiencing only grade 1 mucositis (*p* < 0.001). Notably, some participants in the coconut oil group did not develop mucositis at all throughout radiotherapy (*p* = 0.001). Furthermore, coconut oil improved mucositis grades during subsequent weeks (24.1%, *p* < 0.001).

Additionally, significant improvements were observed in associated complications of radiation-induced oral mucositis such as pain (*p* < 0.001) and trismus Table 7 (*p* = 0.006) along with overall enhancement in quality of life (*p* = 0.009).

To date, no single intervention has been able to fully prevent or treat oral mucositis. Importantly, no prior studies in the literature have reported on the use of coconut oil pulling as an anti-inflammatory agent for radiation-induced oral mucositis. In our study, none of the participants reported any side effects associated with coconut oil use.

**Table 7:** Number of patients showing the onset of trismus in both the groups

	1st week	2nd week	3rd week	4th week	5th week	6th week	Follow up
Treatment	0	0	1 (1.6%)	7 (11.2%)	9 (14.5%)	9 (14.5%)	12 (19.3%)
Control	0	3 (4.8%)	9 (14.5%)	14 (22.6%)	18 (29.0%)	22 (35.4%)	16 (25.8%)
*X*2	0	–	7.089	2.929	3.992	7.486	0.816
*P*		0.892#	0.0077**	0.0869	0.0457	0.0062**	0.366

^#^-Fishers exact test

^**^Highly significant

## Conclusion

The current investigation unequivocally proves that coconut oil pulling is a feasible, safe and highly effective natural remedy for both preventing and alleviating radiation-induced oral mucositis. Its proven anti-inflammatory, antifungal, antimicrobial, and analgesic properties provide significant therapeutic benefits in managing radiation-induced oral mucositis and reduce the severity of its associated complications.
